# Supplementary material for: Evidence supported by Mendelian randomization: impact on inflammatory factors in knee osteoarthritis
Source: Front Med (Lausanne). 2024 May 28;11:1382836. doi: 10.3389/fmed.2024.1382836 (PMC11165061; doi:10.3389/fmed.2024.1382836)
Supplement: Supplementary file 1 [file Data_Sheet_1.ZIP › data/GCST90274802_5e6/802.forest.pdf]

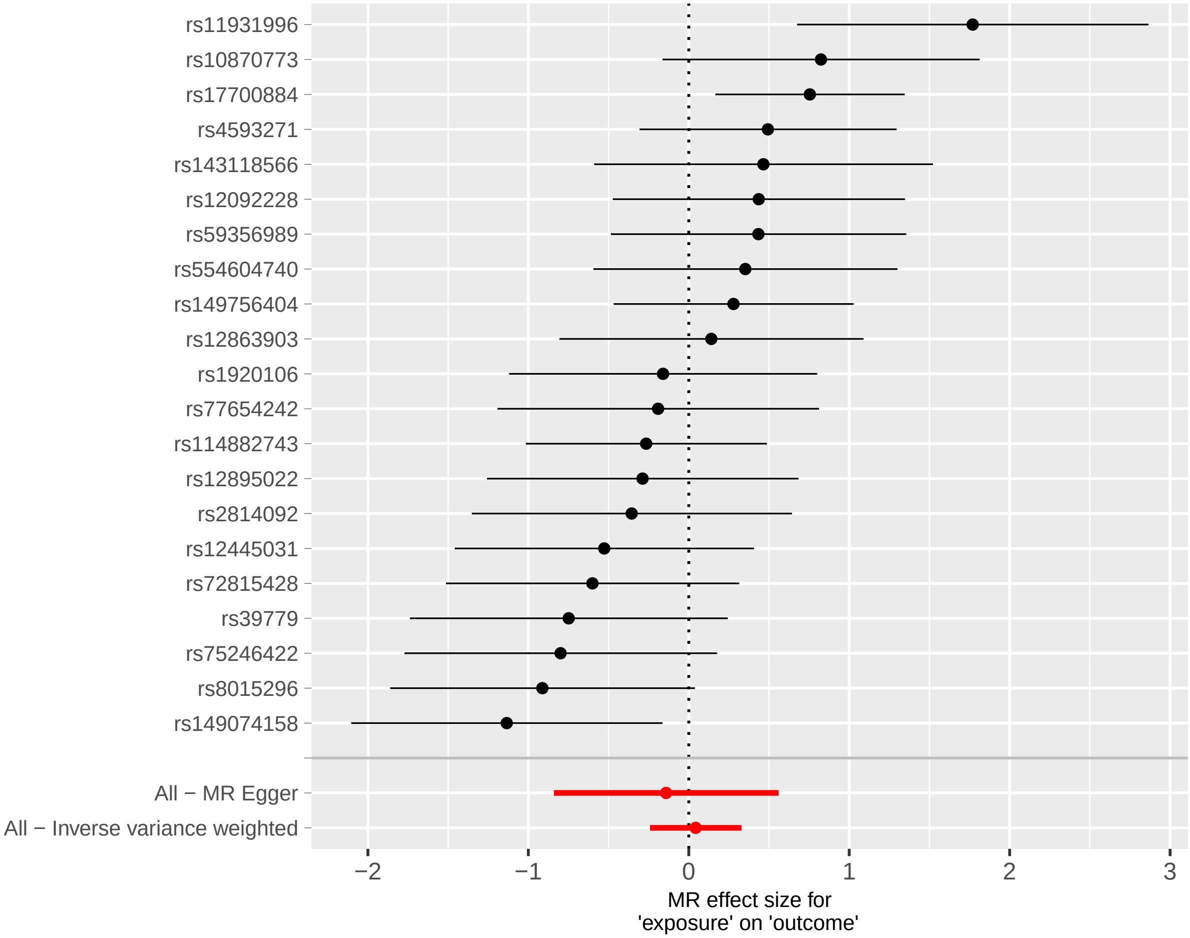
rs11931996
rs10870773
rs17700884
rs4593271
rs143118566
rs12092228
rs59356989
rs554604740
rs149756404
rs12863903
rs1920106
rs77654242
rs114882743
rs12895022
rs2814092
rs12445031
rs72815428
rs39779
rs75246422
rs8015296
rs149074158
All − MR Egger
All − Inverse variance weighted
−2
−1
0
1
2
3
MR effect size for
'exposure' on 'outcome'
